# Feasibility of Breast Cancer Metastasis Assessment of Ex Vivo Sentinel Lymph Nodes through a p-H&E Optical Coherence Microscopic Imaging System

**DOI:** 10.3390/cancers14246081

**Published:** 2022-12-10

**Authors:** Sey-En Lin, Wei-Wen Chang, Ping-Kun Hsiao, Mao-Chih Hsieh, Wei-Yu Chen, Chia-Lang Fang, Chien-Chung Tsai

**Affiliations:** 1 Department of Anatomic Pathology, New Taipei Municipal Tucheng Hospital (Built and Operated by Chang Gung Medical Foundation), New Taipei City 23652, Taiwan; 2 Department of Pathology, Taipei Medical University Hospital, Taipei Medical University, Taipei 11031, Taiwan; 3 Department of Pathology, Wanfang Hospital, Taipei Medical University, Taipei 11696, Taiwan; 4 Division of General Surgery, Department of Surgery, Wanfang Hospital, Taipei Medical University, Taipei 11696, Taiwan; 5 AcuSolutions Inc., 3F., No. 2, Ln. 263, Chongyang Rd., Nangang Dist., Taipei 11573, Taiwan

**Keywords:** OCM, OCMIS, pseudo-H&E, virtual H&E, optical section, sentinel lymph node, metastasis, breast cancer, IDC, ILC

## Abstract

**Simple Summary:**

Pseudo-H&E (p-H&E) imaging using a fluorescence-in-built optical coherence microscopic imaging system (OCMIS) is a novel microstructural technique for assessing en face optical sections of ex vivo specimens. In this study, the comparison of p-H&E, frozen-sectioned H&E, and paraffin-sectioned H&E images was intraoperatively realized through ex vivo sentinel lymph node (SLN) biopsy for breast cancer metastasis assessment. The results revealed that OCMIS is a useful tool for intraoperative breast cancer metastasis assessment through SLN biopsy in patients requiring surgery.

**Abstract:**

Frozen-sectioned hematoxylin–eosin (H&E) image evaluation is the current method for intraoperative breast cancer metastasis assessment through ex vivo sentinel lymph nodes (SLNs). After frozen sectioning, the sliced fatty region of the frozen-sectioned specimen is easily dropped because of different freezing points for fatty tissues and other tissues. Optical-sectioned H&E images provide a nondestructive method for obtaining the insight en face image near the attached surface of the dissected specimen, preventing the freezing problem of fatty tissue. Specimens from 29 patients at Wanfang Hospital were collected after excision and were analyzed at the pathology laboratory, and a fluorescence-in-built optical coherence microscopic imaging system (OCMIS) was then used to visualize the pseudo-H&E (p-H&E) images of the SLNs for intraoperative breast cancer metastasis assessment, and the specificity, sensitivity, and accuracy were 100%, 88.9%, and 98.8% (*n* = 83), respectively. Compared with gold-standard paraffin-sectioned H&E images, the specificity, sensitivity, and accuracy obtained with the frozen-sectioned H&E images (*n* = 85) of the specimens were the same as those obtained with the p-H&E images (*n* = 95). Thus, OCMIS is a useful noninvasive image-assisted tool for breast cancer metastasis assessment based on SLN images.

## 1. Introduction

Biopsy of the sentinel lymph node (SLN) in breast cancer (BC) is the gold standard for assessing the status of lymphatic metastasis [1], which guides intraoperative and chemotherapeutic treatment. At present, most patients undergo minimally invasive surgery to avoid BC-related lymphedema and nerve damage due to axillary lymph node dissection [2]. In invasive breast carcinoma, metastasis occurs through the lymphatic circulation, initiating from the axillary lymph nodes [3,4]. Therefore, intraoperative histopathological analysis of the SLN for assessing BC metastasis [5] can help surgeons decide whether to perform partial axillary lymph node dissection [6], which would substantially improve the patient’s quality of life.

To date, the only procedure for the rapid intraoperative assessment of BC-SLN metastasis is frozen-section (FS) analysis [7], in which 4 μm-thick FS sections of the intraoperatively dissected SLN with hematoxylin–eosin (H&E) staining are sent for immediate histopathological analysis to check for the presence of metastatic cancer cells. However, H&E-stained FS slices have the following problems [8,9]: (1) ice crystal damage and a high water percentage in tissues after high-rate freezing; (2) loss of most fat tissue due to the different freezing point of general tissues (approximately −20 °C); (3) overlapped folding position of a slice, resulting in blurry and dark H&E images; (4) scratching and stretching patterns, resulting from protrusions of the blade; and (5) too heavy or too light staining caused by membrane damage during the freezing process. The most challenging aspect is that the capsule of the SLN is usually not completely sliced because of loss of the capsule boundary caused by the loss of fat tissue [10,11], leading to false-negative results [12,13,14].

Using fresh tissues for rapid histopathological assessment through an optical sectioning technique, such as optical coherence tomography (OCT) [15,16], can not only overcome all the aforementioned problems with FS analysis but also preserve the original morphology of native tissue for further tissue processing (such as immunohistochemistry (IHC) and polymerase chain reaction). Moreover, the steps of frozen mounting and physical slicing are skipped to shorten the histopathological assessment time. Conventional OCT can provide sufficient depth penetration (1–2 mm) for tissue scans, but the spatial resolution (~5 μm) is insufficient for tissue assessment. Twenty years ago, full-field OCT (FF-OCT) was proposed to improve the spatial resolution [17,18], thereby increasing the level of detail in tissue morphology, but the nucleus morphology could not be assessed, which is critical for differentiating between normal and cancer cells [19]. In 2015, FF-OCT was used to assess the BC metastasis of SLN [20] in grayscale, but the lack of nuclear details caused a very high false-positive rate (~17%). Typically, the specificity and sensitivity of intraoperative SLN assessment are approximately 100% and 85%, respectively. To improve accuracy, nuclear information is required [21]. In 2019, a study analyzed thin-sliced formalin-fixed and paraffin-embedded (FFPE) lymph node sections using label-free deep-ultraviolet excitation fluorescence microscopy, which is similar to microscopy with ultraviolet surface excitation (MUSE), and proposed two channels to represent cytoplasmic and nuclear patterns, but this technique could capture only surface patterns [22]. Stimulated Raman scattering microscopy is a label-free method for obtaining cytoplasmic and nuclear images based on the protein and lipid channels [23], but the produced two-channel images present nuclear patterns that make it challenging to differentiate between cytoplasmic and nuclear contents. In 2020, FF-OCT with label-free dynamic cell imaging (DCI) was used to enhance assessment of the nucleus [24], but the specific response of the nuclei to DCI was still insufficient due to the different elastic vibrations of different cell types. Furthermore, the outputs of all these modalities do not resemble that of H&E-like processing, which increases the learning time of pathologists for the assessment of intraoperative specimens. To decrease pathologists’ learning curve, the resulting images need to be color-coded similarly to those produced by H&E-like staining [25]. In 2009, the virtual H&E color-coding method from the source inputs of fluorescence-only (nucleus) and reflectance-only (cytoplasm) channels was first used [26]. To improve the semitransparent effect of absorptive dyes, the Beer–Lambert law was implemented in virtual H&E processing [27].

In this proof-of-concept study, we used an in-house-developed optical-sectioning method, namely, the optical coherence microscopic imaging system (OCMIS) [28] combining FF-OCT (or so-called optical coherence microscopy, OCM) with built-in fluorescence microscopy (FM), to provide pseudo-H&E (p-H&E) images [29] for intraoperative assessment. The p-H&E imaging features of the SLN on co-located slides were validated against those of images with H&E staining [30]. This imaging modality provides <1 µm isotropic spatial resolution for tissues, and the resulting images preserve the fundamental details of all relevant structures, such as normal cells, malignant clustered cells, fibrosis, adipocytes, veins, and arterioles. These properties decrease the false-negative rate, and more excised tissues are preserved because of the lack of FS-related waste.

## 2. Materials and Methods

### 2.1. Study Participants

SLN biopsy of BC was initiated in 2006 at Wanfang Hospital [31], Taipei, Taiwan. From 2006 to 2015, 686 women were treated surgically for BC, including ductal carcinoma in situ (DCIS), of whom 656 underwent surgery first and 30 received neoadjuvant chemotherapy. The SLNs of all patients were excised by reference to axillary reverse mapping (ARM) [32]. In 1996, a dual tracer (technetium-labeled sulfur colloid [33] and vital blue dye [34]) was used to excise SLN [35], and this dual tracer remains the current standard [36]. In 1997, patent blue V [37] was used to trace breast SLNs. Ten years before, methylene blue (MB) dye gradually became a popular and acceptable blue dye for this surgery [38]. In 2017, a combination of MB and indocyanine green (ICG) [39] was assessed to preserve the advantages of MB while facilitating ARM. Some recent studies have applied only ICG [40] in ARM and tumor surgeries, but ICG alone cannot distinguish tumors from inflammation. In this study, breast SLNs were identified using patent blue V injections because of the high application experience and low toxicity.

Following the established protocols in Wanfang Hospital, we examined ex vivo SLNs with suspected BC metastasis resected intraoperatively during lumpectomy and mastectomy from 29 patients (age range: 34–87 years) admitted to the Department of Surgery over 6 months. Of the resected SLNs, tumor laterality was left in 16 patients and right in 13 patients. Their final diagnoses were invasive ductal carcinoma (IDC) (*n* = 18; stage IA: 11, IIA: 2, and IIB: 5), DCIS (*n* = 7), invasive lobular carcinoma (ILC; *n* = 3, stage IIA: 2 and IIB: 1), and lobular carcinoma in situ (LCIS, *n* = 1). All participants (or their relatives) signed a written consent form, and this study was approved by the Institutional Review Board.

### 2.2. Study Design

**Figure 1:**
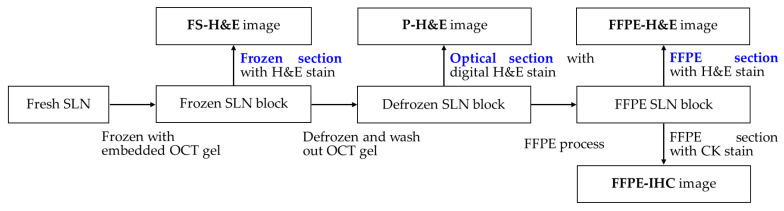
Processing flow of an ex vivo SLN for the production of FS-H&E, p-H&E, FFPE-H&E, and FFPE-IHC images.

To prevent our experiment from interfering with the timely intraoperative assessment of SLN metastasis, an ex vivo scanning process of OCMIS was performed only after the regular FS analysis. FS-H&E results were assessed by three pathologists (SEL, CLF, and WYC) in a blinded manner, and the p-H&E images obtained using OCMIS were evaluated by an experienced pathologist (SEL) in a blinded manner. FFPE-H&E and FFPE-IHC analyses were performed as per the routine diagnostic procedure of the hospital. Finally, we compared the findings from OCMIS scans with the FS-H&E, FFPE-H&E, and FFPE-IHC results. Figure 1 illustrates a flowchart of ex vivo SLN processing.

### 2.3. Tissue Processing and OCMIS Image Acquisition

**Figure 2:**
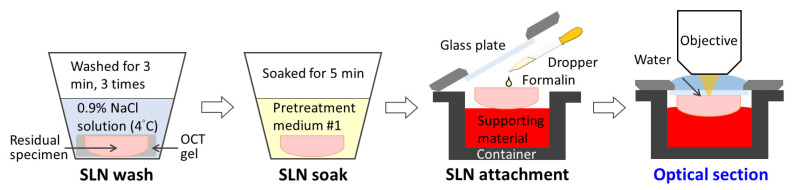
Ex vivo SLN processed from the end of FS to the OCMIS.

All excised ex vivo SLNs were processed according to Figure 1. First, SLNs were transferred from the operating room to the pathological department, where the fresh SLNs were dissected and processed into frozen SLN blocks by embedding the samples into optimal cutting temperature gel (OCTG). The samples were frozen (at approximately −20 °C), sliced, and stained with H&E. Next, the remaining frozen SLN blocks were thawed, and the OCTG was washed out with 0.9% NaCl solution for 9 min (three times for 3 min each; Figure 2). This step is necessary because the residual gel can affect p-H&E performance. In the hospital, a routine FFPE-IHC cytokeratin (CK) stain is used to detect metastatic tumors after an FFPE-H&E image is negative for malignancy. As presented in Figure 2, the single defrozen SLN block was soaked in AcuSolutions pretreatment medium (PM) #1 for 5 min, PM #1 having a Hoechst 34580 concentration of 50 μg/mL (dissolved in 1× PBS). Owing to the space limitations of the tissue container, the acceptable tissue size was less than 2 cm × 2 cm × 0.5 cm. After SLN soaking, the residual SLN was lightly placed on the supporting material in the container, and 10% neutral phosphate-buffered formalin (Leica Surgipath, Leica Biosystems Richmond, Inc., Richmond, IL 60071, USA) was dropped on the surface of the residual SLN for SLN attachment. Without formalin, SLN attachment would be poor, especially when a specimen has a high fat content. Finally, the specimen-containing container was placed inside the AcuOnPath instrument to generate p-H&E images.

The OCMIS (AcuOnPath, AcuSolutions, Taipei, Taiwan) [30] can produce p-H&E images by stitching multiple side-by-side fields at a 15 μm depth beneath the surface of the residual SLN being scanned. After a surface snapshot of the container and optical section was obtained, a large-field p-H&E image of the residual SLN was generated, and the scanned field could be cropped through the viewer interface (AcuViewer, AcuSolutions).

As presented in Figure 1, the scanned residual SLN was immediately placed in 10% neutral phosphate-buffered formalin overnight and processed as an FFPE block. A slice of the FFPE section was taken for H&E staining. After the SLN assessment of FS, the pathologist decided whether to obtain an additional slice for FFPE sectioning (Figure 1) to perform CK IHC staining for the identification of metastatic tumor cells, for instance, when FS-H&E images were negative or suspicious.

**Figure 3:**
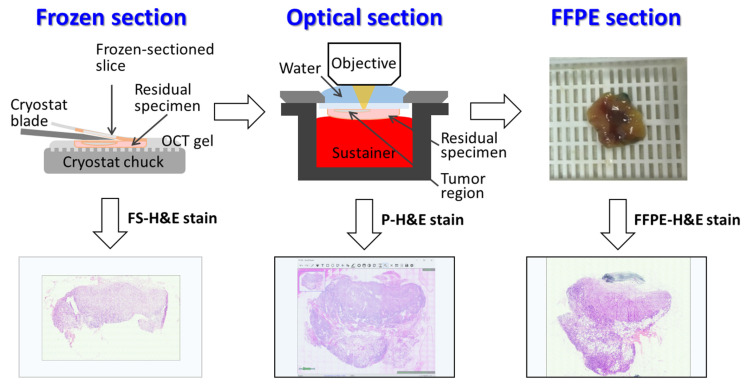
Three pathways—frozen section (FS), optical section, and formalin-fixed paraffin-embedded (FFPE) section—were used for the same raw specimen in a tissue processing series. With FS-H&E, p-H&E, and FFPE-H&E stains, three individual images were generated.

Figure 3 presents a comparison of the images obtained using FS, OCMIS, and FFPE for an ILC case with SLN metastasis. Both FS-H&E and FFPE-H&E slides of the sectioned SLN were scanned using a whole-slide scanner (NanoZoomer-SQ, Hamamatsu, Photonics K.K., Hamamatsu, Japan) and examined using an image viewer (NDP.view2, Hamamatsu Photonics K.K.). Unlike FS-H&E and FFPE-H&E slides, the optical section in OCMIS uses a digital stain for stitched images.

### 2.4. Ex Vivo Fluorescence-in-Built OCMIS

The p-H&E images generated by AcuOnPath G-AP-001 (AcuOnPath, Taipei, Taiwan) were evaluated using a pair of water immersion objectives (magnification: 16×, numerical aperture: 0.8) [30]. Two LED light sources with central wavelengths of 385 nm (near ultraviolet) and 590 nm (orange) were used for the excitation of nuclei and the interference pattern of the cytoplasm, respectively. Both nuclear and cytoplasmic images were acquired alternatively by a CMOS camera. Hoechst 34580 (performance equivalent to Hoechst 33342), a live-cell DNA dye for nuclei, was excited by a near-ultraviolet light source (fluorescence mode) to visualize nuclear structures. The orange light source was used to generate the interference signal (interference mode) for the cytoplasmic structures [28]. The images from the fluorescence and interference channels were independently saved as gray values in two separate memories [29]. The fields of view (FOVs) from both the fluorescence and interference channels were 0.9 × 0.7 mm^2^. With the built-in color-coding algorithm, the nuclei and cytoplasm were presented with a yellow-and-deep-green color scheme with a black background and then converted to a pink-and-blue-violet color scheme with a white background in the produced p-H&E image. With a high spatial resolution for tissues via this modality [30], high-quality p-H&E images were produced. The p-H&E images provided a similar level of detail to conventional histology and had good feasibility with respect to the intraoperative evaluation of ex vivo SLN metastasis of BC.

### 2.5. Histological Classification

According to the UICC guidelines, SLN metastases were classified as follows: (1) macrometastatic lesion (diameter > 2 mm); (2) micrometastatic lesion (diameter: 200 µm to 2 mm); and (3) isolated tumor cells (ITCs, diameter < 200 µm). The number of isolated lymph nodes, number of metastatic lymph nodes, and size of the metastases (micrometastatic or macrometastatic) were recorded during ARM. SLNs of ARM, including macro- or micrometastases, were considered to be positive cases, whereas those including ITCs and/or nonmetastasis were considered to be negative. The accuracy, specificity, and sensitivity in this study represent the performance of BC metastasis assessment of intraoperative SLNs through OCMIS.

### 2.6. Data Collection and Statistical Analysis

SLN biopsies were classified as false positive (FP), false negative (FN), true positive (TP), or true negative (TN). Specificity was defined as (TN)/(TN + FP). Sensitivity was defined as (TP)/(TP + FN). Accuracy was defined as (TP + TN)/all cases.

## 3. Results

**Figure 4:**
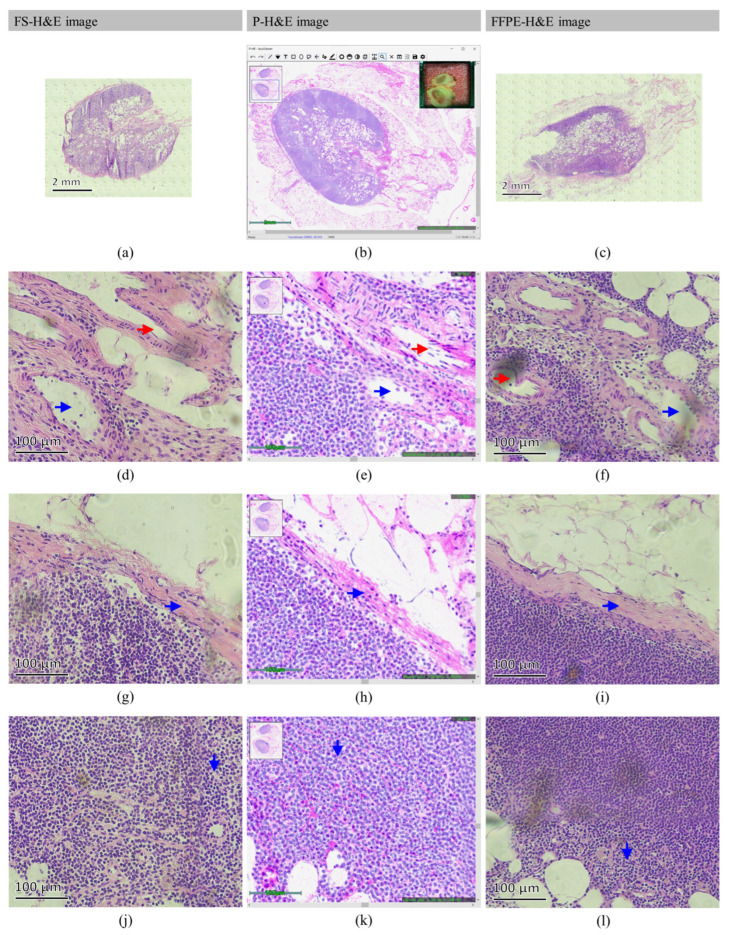
Comparison of FS-H&E, p-H&E, and FFPE-H&E images acquired from the same normal SLN #A of patient #6. (**a**–**c**) present the images of the lower specimen indicated in the true-tissue snapshot (top-right inset) of (**b**); the top-left inset of (**b**) is the browsing navigator. In (**d**–**f**), red and blue arrows indicate arterioles and veins, respectively. Blue arrows in (**g**–**i**) indicate the capsule between the fat and cortex. Blue arrows in (**j**–**l**) represent the lymphatic vessels in the cortex region.

**Figure 5:**
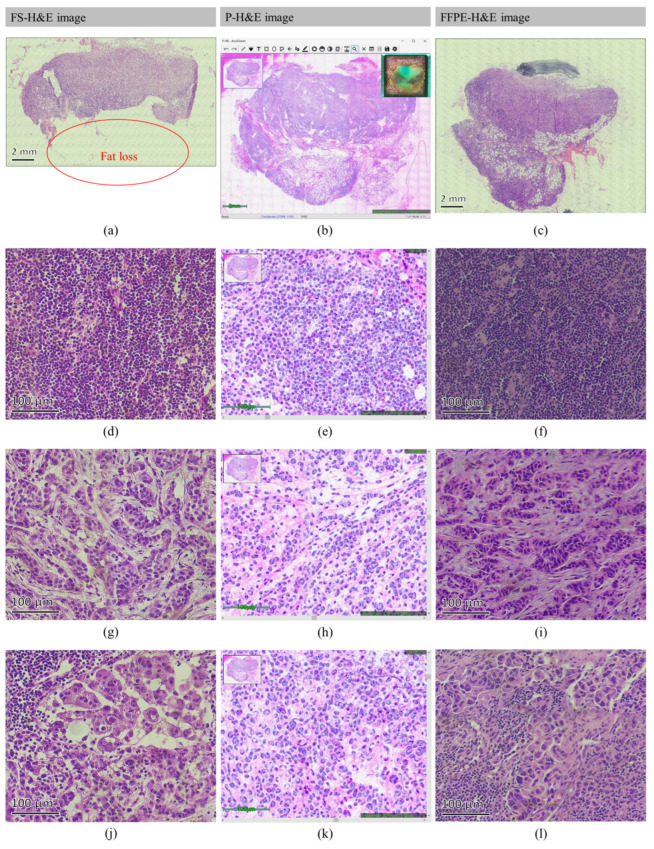
Comparison of FS-H&E, p-H&E, and FFPE-H&E images acquired from the same IDC-metastatic SLN #A1 of patient #28. (**a**–**c**) present the images of the true-tissue snapshot (top-right inset) of (**b**); the top-left inset of (**b**) is the browsing navigator. In (**d**–**f**), lymphocytes are crowdedly distributed in all figures. (**g**–**i**) display the poorly formed glandular morphology of cancerous aggregation. (**j**–**l**) show disordered growths with poor differentiation in morphology.

Figure 4 and Figure 5 present the acquired FS-H&E, p-H&E, and FFPE-H&E images of a normal and metastatic SLN, respectively. To clearly illustrate the features, only the representative images from the patients are presented.

In Figure 4, a biopsied SLN from patient #6 was bisectioned to be placed in the central and lower positions of the glass plate. In Figure 4b, the top-right inset displays the true-specimen snapshot after cropping the scanned area in the OCMIS p-H&E image. The top-left navigator depicts the browsing area (cropped in a blue-line rectangular box) of the whole scanned field for zoom-in or zoom-out conditions through the in-house developed AcuViewer interface [30]. This shows that all the surrounding fat tissues were completely preserved. Compared with Figure 4a,c, the overall appearance is the same, but Figure 4a,c exhibits folding, scratching, stretching, and tissue-loss problems. Figure 4e displays the arterioles (red arrow) and veins (blue arrow) in the vessel-rich region of Figure 4b,d,f, which present the corresponding FS-H&E and FFPE-H&E images of the same specimen. Here, the orthogonal patterns of the arteriolar nuclei in Figure 4d–f are all obvious. As presented in Figure 4h, the capsule (blue arrow) between the cortex and fat is obtained, and Figure 4g,i exhibit good correspondence with Figure 4h. However, the fat in Figure 4g is lost because the freezing points of general and fat tissues are different and because fat tissues are easily lost during frozen sectioning. In this SLN, as presented in Figure 4k, a lymphatic vessel (blue arrow) is found where the same structures (blue arrows) in Figure 4j,l are not easy to observe because lymphatic vessels are not easy to stain.

In Figure 5, a biopsied SLN from patient #28 was also bisectioned, and only half of the specimen (SLN #A1) was placed under the glass plate. Referring to Table 1, patient #28 was an IDC case. As presented in Figure 5a, the fat-rich region was lost due to poor attachment during frozen sectioning. By contrast, Figure 5b,c represent the complete contours for further assessment. Figure 5e depicts the lymphocyte-rich area zoomed in from the cortex region of Figure 5b,d,f, which confirm the cortical morphology crowded with lymphocytes, but the cell density in Figure 5d,e is not as high as that in Figure 5f. In this intraoperative SLN biopsy (the true-specimen snapshot in Figure 5b), the blue-green spot is the location of the injected patent blue V dye [37] used to visualize the SLNs. These dyes do not affect the scan performance of OCMIS because of the low concentrations of patent blue V dyes with low optical absorption. Figure 5h displays the Indian file growth pattern of the ILC-like structure, and Figure 5g,i present poorly formed gland structures with small and condensed nuclear aggregations. Figure 5k accentuates huge and poorly differentiated IDC glands, and Figure 5j,l present the ice-crystal-destroyed cavities from the frozen sectioning process and ethanol-dehydrated cracks during tissue processing for FFPE.

### 3.1. Normal SLN

**Figure 6:**
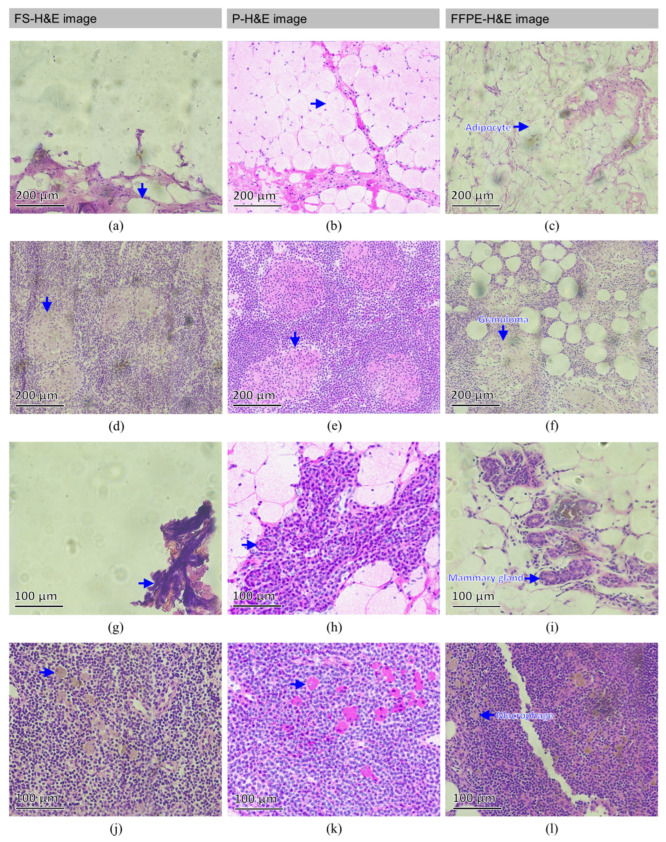
Featured positions of FS-H&E, p-H&E, and FFPE-H&E images acquired from the same normal SLNs of individual patients. Blue arrows in (**a**–**c**) indicate the fat areas from the same normal SLN #B of patient #6. Blue arrows in (**d**–**f**) indicate the plural granulomatous regions in the cortex of the same normal SLN #A1 of patient #7. In (**g**–**i**), the mammary glands (blue arrows) are sectioned with ductal morphology from the same normal SLN #B of patient #11. In (**j**–**l**), many macrophages (blue arrows) can be seen to have gathered at certain positions from the same normal SLN #A of patient #6.

In Figure 4, a full-vision comparison of FS-H&E, p-H&E, and FFPE-H&E images from the same normal specimen is provided. In detail, some featured structures are found in these normal SLNs, as presented in Figure 6.

Figure 6b displays the complete fat tissue distribution with the original adipocytes from the normal SLN #B of patient #6. The blue arrow indicates a single adipocyte with an intact boundary surrounded by other adipocytes. By contrast, Figure 6a depicts the fat loss caused by frozen sectioning in most of the region adjacent to that shown in Figure 6b. As a clearer image than Figure 6a, Figure 6c depicts no fat loss, but all adipocytes exhibit considerable shrinkage. Figure 6e reveals plural granulomatous regions in the cortex of the SLN #A1 of patient #7. In comparison, Figure 6d,f indicate the same pathological features in the adjacent regions of the same specimen. In Figure 6d–f, circular clusters indicated by blue arrows are granulomas typically induced by inflammation. Figure 6h presents the mammary glands sectioned with ductal morphology from the SLN #B of patient #11, where the boundary of the duct is clear. In Figure 6g, the mammary glands surrounded by fat tissue are destroyed by frozen sectioning; thus, the structure stained with H&E is blurry and unrecognizable. In Figure 6i, although the mammary gland is complete, the surrounding fat tissue still shows shrinkage, and the mammary gland structure is disrupted. In SLN #B of patient #11, there are only normal mammary glandular cells and adipocytes found in the p-H&E image (arterioles and veins are easily identified and are not considered in this image). In the H&E image of FFPE of the same specimen, SLN #B of patient #11, only normal mammary glandular cells and adipocytes were found (arterioles and veins can be easily identified and were not considered in this image). So, we were able to ensure confirmation of the validations of both normal mammary glandular cells and adipocytes. In Figure 6k, many macrophages (blue arrows) were found in the SLN #A of patient #6, where the cytoplasm is colorized as magenta due to the high reflected intensity of OCM. By contrast, the macrophages in Figure 6j,l are all khaki in color. Although the colors of the macrophages in Figure 6k are not the same as those in Figure 6j,l, the cells can still be easily visualized due to the high-contrast morphology. Based on the information that had been confirmed for SLN #A of patient #6, as the familiar adipocytes, arterioles, veins, capsules, and lymphocytes were excluded, the rest of the featured cells in the FFPE-H&E image of SLN #A of patient #6 were identified as macrophages on the basis of the pathologist’s experience. For the same tissue of SLN #A of patient #6, the rest of the featured cells were also found in the scanned p-H&E image. So, we can say that these cells are macrophages. In addition, the cytoplasmic and nuclear sizes of the macrophages in the p-H&E image fit the properties of the FFPE-H&E image for the same tissue specimen. So, macrophages were also confirmed.

### 3.2. Metastatic SLN

**Figure 7:**
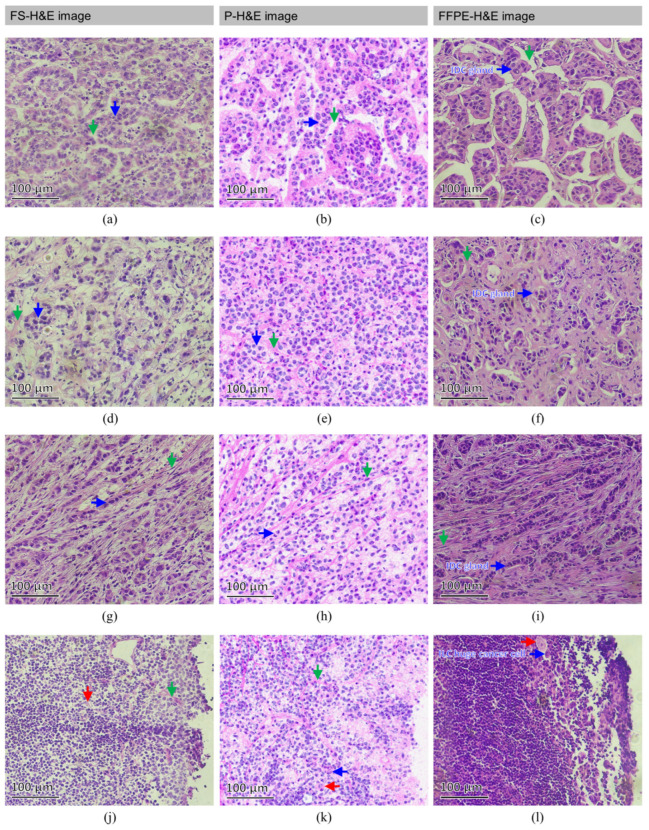
Featured FS-H&E, p-H&E, and FFPE-H&E images of the equivalent positions acquired from the same BC-metastatic SLNs of individual patients. Blue arrows in (**a**–**c**) indicate the invasive glandular cells from the same IDC-metastatic SLN #A of patient #24, while green arrows indicate the cleft spaces enhanced by frozen fracture and FFPE dehydration. The blue arrows of (**d**–**f**) indicate the invasive glandular regions (transversal section) from the same IDC-metastatic SLN #A of patient #23, while green arrows indicate stromal fibrosis. Blue arrows in (**g**–**i**) represent the invasive ductal carcinoma with Indian file pattern (longitudinal section) from the same IDC-metastatic SLN #A2 of patient #28, while green arrows indicate the stromal fibrosis as well. In (**j**–**l**), all the right regions from the same ILC metastatic SLN #A of patient #11 indicate atypical morphology: (1) green arrows indicate the stromal collagen fibers; (2) red arrows indicate the benign stromal macrophages; (3) blue arrows indicate the metastatic ILC pleomorphic enlarged atypical cancer cells. In (**j**), no atypical cancer cells were found.

In Figure 5, a full-vision comparison of FS-H&E, p-H&E, and FFPE-H&E images from the same BC-metastatic specimen is presented. In detail, some featured structures were found in these normal SLNs, as presented in Figure 7. The color variations in the FS-H&E and FFPE-H&E images are obviously high, but the p-H&E images were still very uniform and homogeneous when all the images were processed at different times.

Figure 7b displays the invasive glandular cells from the IDC-metastatic SLN #A of patient #24. In Figure 7b, the IDC pattern (blue arrow) is arranged and separated by a space (green arrow). Figure 7a,c are FS-H&E and FFPE-H&E images of the areas adjacent to that shown in Figure 7b from the same SLN specimen. Figure 7e represents the transversal-sectioned invasive glandular cells from the IDC metastatic SLN #A of patient #23, where the IDC pattern (blue arrow) is arranged and separated by surrounding stromal fibers (green arrow). With surrounding stromal fibrosis, the native structure and tactile are solid and rigid. In Figure 7d, the structure is loose and damaged due to the frozen sectioning procedure. In Figure 7f, the splitting gap between glandular cells and stromal fibrosis is formed by FFPE-processing shrinkage. By contrast, in Figure 7e, the raw morphological pattern is preserved. Figure 7h displays the longitudinal-sectioned invasive glandular cells from the IDC metastatic SLN #A2 of patient #28, where the IDC pattern (blue arrow) is arranged as an Indian file pattern [41] and surrounded by stromal fibers (green arrow). This longitudinal Indian file pattern in IDC is similar to the ILC-specific pattern, and pathologists can misjudge the disease based on this pattern. Figure 7g,i show the FS-H&E and FFPE-H&E images of the areas adjacent to that shown in Figure 7h from the same SLN specimen. Figure 7k displays the atypical morphology from the ILC metastatic SLN #A of patient #11; the green arrow indicates the stromal collagen fibers, the red arrow indicates the benign stromal macrophages, and the blue arrow indicates the metastatic ILC pleomorphic enlarged atypical cancer cells [42]. All the properties of Figure 7k are included in Figure 7l, and no atypical cancer cell can be found in Figure 7j. Therefore, this is a missed case of ITCs in SLN for FS-H&E in this trial.

### 3.3. Processing Time Comparison

To validate the performance of the processing of the p-H&E images, FS-H&E images from the same SLN biopsy were compared for rapid intraoperative assessment. In this trial, 29 patients with 2.9 SLNs on average were tested in term of FS-H&E and p-H&E processes. In a BC SLN study in 3303 patients that was conducted between 2015 and 2019, the mean SLN number was 3.6 ± 2.6 [43], and this number is higher than that in our trial. In Table 2, the processing items for producing FS-H&E and p-H&E images are listed. To generate FS-H&E images (FS-H&E slides), the total processing time for a patient is 45 min on average, including tissue dissection, tissue processing, and image assessment. By contrast, to generate p-H&E images, the total processing time for a patient is 56 min on average, including the PM #1 soak, specimen setting, specimen scanning, image processing, and image assessment. The total specimen scanning time for a patient on average is 11.5 × 2.9 = 33.4 (min). The p-H&E assessment is 8 min shorter than the FS-H&E assessment.

### 3.4. Data Assessment

In Table 3, the SLN parameters and assessments for the FS-H&E, p-H&E, and FFPE-H&E images are listed, and the SLN numbers for the FFPE-H&E images are higher than those for the FS-H&E and p-H&E images. The reason is that the SLNs dissected for FS were obvious and apparent due to the dissection having been conducted in a limited time; thus, the residual tissues after FS will be sent to further pick out additional SLNs as FFPE-H&E references.

The statistical parameter in this trial is the SLN number, *n*. According to the statistical criteria set out in Section 2.6, the TP, TN, FP, and FN rates for the FS-H&E and p-H&E images were verified. Furthermore, the specificity, sensitivity, and accuracy of both the FS-H&E and p-H&E images were 100%, 88.9%, and 98.8%, respectively.

## 4. Discussion

Combining OCM and FM methods, OCMIS provides a new sectioning technology in optics with the pseudo-H&E color-coding method. Without any physical slicing tissue wastage, this optical-sectioning technology has the highest tissue conservation rate, as it can conserve an intact raw SLN specimen for further processing, for instance, in FFPE analysis or genetic testing. During the intraoperative process, currently, the only way to make a rapid assessment of a metastatic SLN is frozen sectioning with H&E staining, and this method requires physical slicing after receipt of the fresh specimen chunk, but physical slicing after frozen sectioning has many problems, including fat loss, ice crystal damage, scratching, stretching, multiple folding, etc. In contrast, our technology can provide H&E-like images of fresh tissue chunks using OCMIS by optical sectioning without any physical slicing, so the traditional freezing step can be skipped.

Space utilization and profit from medical devices are two key ways of generating income for a hospital or medical center. All the parts of the AcuOnPath instrument can be integrated on a double-layer desk with a setting area of less than 60 cm × 100 cm [30]. This operating space is much smaller than that required for conventional FS-H&E processing equipment. Furthermore, the machine can be placed on a movable desk to ensure its proximity to the intraoperative or rapid on-site evaluation (ROSE) [44,45] room. Presumably, given that the assessment results are noninferior to those of FS-H&E assessment, hospitals and medical centers will be willing to use it, as the space and movement problems are solved. The feasible applications are not limited to intraoperative BC-SLN metastasis.

The FS protocol is complicated in comparison with that for producing p-H&E images from raw SLN specimens. In addition, a cryostat requires experienced and skilled technicians and sophisticated equipment to obtain the best FS-H&E slides of SLNs for daily operations, which might lead to problems for hospitals or medical centers when an irreplaceable operator is absent. By contrast, AcuOnPath is easy to operate. With only four easy steps (i.e., immersion, setting, taking a snapshot, and scanning), p-H&E images of raw SLN specimens can be produced and then examined. Furthermore, no tissue wastage occurs when obtaining p-H&E images, which is very important as the tissues are small and limited.

FS-H&E imaging has many problems, such as the loss of fat tissue. Moreover, metastatic cancer cells typically invade SLNs from capsules, resulting in a high false-negative rate, as the fat, capsules, and metastatic cancer cells drop together. In addition, SLNs of patients after neoadjuvant chemotherapy are easily deformed and destroyed after being frozen, making it difficult for pathologists to judge malignant metastasis [46]. Using AcuOnPath, these freezing problems can be avoided because there is no freezing process.

Given the sufficient pixel and spatial resolutions of OCMIS with AcuOnPath, many structural details of normal and metastatic SLNs were observed. Using the established featured patterns of the image database (referring to Figure 4, Figure 5, Figure 6 and Figure 7) in this trial, the pathologist easily judged the SLN status of the patient. Generally, p-H&E images were consistent with FS-H&E images of the same specimen. After pathological assessment, the specificity, sensitivity, and accuracy of both the p-H&E and FS-H&E images were all the same. However, one more ITC case was found through p-H&E in the SLNs compared with the FS-H&E method, and this ITC case could highly affect the prognosis of the patient, as chemotherapy was not administered after evaluation. As p-H&E images are derived from two independent image sources, OCM (cytoplasm) and FM (nucleus), OCMIS can provide stable image colors and dual raw data for raw SLN specimens for diagnosis and further AI training.

Regarding processing time, the processing time for the p-H&E method of the BC-SLN trial is longer than that for the FS-H&E protocol. The key to overcoming this limitation is the scan time. If the scan speed of the OCMIS can be doubled, then the processing time for the p-H&E method for this BC-SLN trial will be 41 min, which is better than that for FS. SLN number and SLN size considerably affected the total scanning time for each patient. This proof-of-concept study demonstrated the noninferior results of the BC-SLN trial using the p-H&E method in comparison with the FS-H&E protocol. Collecting more data could help to separate the assessment results for the p-H&E and FS-H&E images. Once double scanning speed with the same image quality is achieved, the next BC-SLN trial can be revised for the proposed processing method [20].

Currently, a series of related methods, such as core needle biopsy of breast cancer for rapid screening assessment during outpatient surgery and intraoperative breast margin during lumpectomy, are being finished and their implementation is being planned. Based on the p-H&E database images collected in this study, these two clinical applications of primary tumor tissues of breast cancers will be of great help in rapid assessment.

## 5. Conclusions

In this proof-of-concept study, an OCMIS-based p-H&E method was used to evaluate intraoperative BC-SLN. The specificity, sensitivity, and accuracy of both the p-H&E and FS-H&E images for the BC-SLN trial were 100%, 88.9%, and 98.8%, respectively, demonstrating the non-inferiority of the p-H&E method to the FS-H&E protocol. Bypassing the freezing and slicing damage of FS, this OCMIS-based p-H&E method provides clear and complete images without any tissue wastage. Without using laser sources for excitation illumination, the OCMIS has high-intensity stability for scanning p-H&E images. Visible-illumination-range OCMIS provides a high spatial resolution for detailed visualization of morphological abnormalities. By using a DNA-specific stain for FM, nuclear images of crowded lymphocytes and high-density tumor glands were clear and distinguishable in the p-H&E images produced in this BC-SLN trial. With digitized color-coding images, it becomes easier to realize remote assessment. Raw 8-bit data from p-H&E images, including those obtained from OCM and FM, can provide individual linear independent inputs for subsequent AI training with better performance and promise to support intraoperative assessment alternatives for pathologists, surgeons, physicians, and hospitals.

## Figures and Tables

**Table 1:** Characteristics of 29 patients with BC whose SLNs were collected for FS biopsy.

Patient	Sex	Age	Laterality	Category			Final Histology	SLN	
				T	N	Stage		Quantity	Mean Size (cm^2^)
1	F	58	L	Tis	N0	0	DCIS	2	1.2 × 0.8
2	F	56	R	Tis	N0	0	LCIS	3	1.2 × 1.0
3	F	60	R	T1c	N0	IA	IDC	5	1.0 × 1.0
4	F	67	R	T1c	N0	IA	IDC	2	0.7 × 0.7
5	F	70	L	Tis	N0	0	DCIS	1	0.8 × 0.8
6	F	49	L	T1b	N0	IA	IDC	3	1.0 × 0.5
7	F	66	R	T1a	N0	IA	IDC	6	N/A
8	F	40	L	Tis	N0	0	DCIS	2	1.0 × 1.0
9	F	42	R	Tis	N0	0	DCIS	2	0.6 × 0.4
10	F	53	L	T1c	N0	IA	IDC	1	1.2 × 1.1
11	F	49	R	T2	N0	IIA	ILC	3	1.4 × 1.4
12	F	73	R	T1mic	N0	IA	IDC	4	1.6 × 1.0
13	F	34	R	T1b	N0	IA	IDC	4	1.2 × 0.7
14	F	66	L	T1c	N0	IA	IDC	5	1.5 × 0.7
15	F	60	R	T2	N0	IIA	IDC	1	0.8 × 0.8
16	F	58	L	T2	N0	IIA	ILC	6	1.5 × 1.5
17	F	68	R	Tis	N0	0	DCIS	2	N/A
18	F	85	L	T2	N0	IIB	ILC	2	1.5 × 1.5
19	F	51	R	T1c	N0	IA	IDC	4	1.8 × 1.5
20	F	49	L	Tis	N0	0	DCIS	2	1.1 × 0.5
21	F	76	L	T2	N1a	IIB	IDC	6	1.8 × 1.8
22	F	48	L	T2	N1mi	IIB	IDC	3	1.9 × 1.9
23	F	87	R	T1c	N1a	IA	IDC	2	0.9 × 0.5
24	F	68	L	T2	N1	IIB	IDC	2	1.5 × 0.8
25	F	48	L	T1c	N0	IA	IDC	3	0.6 × 0.6
26	F	53	L	T2	N0	IIA	IDC	3	1.6 × 1.0
27	F	54	L	T1c	N1a	IIA	IDC	1	3.4 × 1.4
28	F	37	R	T2	N1a	IIB	IDC	2	1.3 × 1.1
29	F	48	L	Tis	N0	0	DCIS	1	1.2 × 0.8
Average		58 ± 13						2.9 ± 1.6	1.3 ± 0.6 × 1.0 ± 0.5

**Table 2:** The processing times for FS-H&E and p-H&E images.

Time (min)	FS-H&E Image	p-H&E Image
Tissue dissection ^1^	4	
PM #1 soak ^2^		5
Specimen setting ^3^		2
Specimen scanning ^4^		11.5
Image processing ^5^		4
Tissue Processing ^6^	29	
Image assessment ^7^	12	8
SLN number ^8^	2.9 ± 1.6	
Total time	45	56

^1^ Tissue dissection is performed to collect and bisect SLNs in delivered biopsy tissue chunks from the surgeon. ^2^ The soaking time for all the SLNs of each patient is 5 min, because all the SLNs can be soaked at the same time. ^3^ Specimen setting includes tissue setting in the container, putting the container in the AcuOnPath, obtaining a tissue snapshot, illumination calibration, compensation calibration, and depth calibration. ^4^ This is the scanning time for a single SLN on average, where each single FOV (0.7 × 0.9 mm^2^) requires 1.8 s to capture OCM and FM images. ^5^ The p-H&E processing time is for the processing of OCM and FM images and combining them to produce p-H&E images without any redundant resolution. ^6^ Tissue processing time for the FS-H&E image is for the freezing, slicing, H&E staining, and mounting protocols. ^7^ The assessment time for FS-H&E images requires additional delivery time from the pathology room to the on-duty pathologist’s office compared with that for p-H&E images. ^8^ The average and standard deviation values of the SLN numbers for each patient.

**Table 3:** Comparison of FS-H&E, p-H&E, and FFPE-H&E assessments of the same specimen.

	FS-H&E Image	P-H&E Image	FFPE-H&E Image
SLN number (*n*)	85	83	95
Positive metastasis	8	8	9
ITCs in SLN ^1^	0	1	4
Positive rate (%)	9.4	9.6	9.5
TP	8	8	9
TN	76	74	86
FP	0	0	0
FN	1	1	0
Specificity (%)	100	100	100
Sensitivity (%)	88.9	88.9	100
Accuracy (%)	98.8	98.8	100

^1^ According to the metastatic criteria of positive assessment, ITCs were not included. However, a patient must be marked as a high-potential metastatic patient for a return visit.

## Data Availability

Data will be made available upon reasonable request.
